# An Introspective Comparison of Random Forest-Based Classifiers for the Analysis of Cluster-Correlated Data by Way of RF++

**DOI:** 10.1371/journal.pone.0007087

**Published:** 2009-09-18

**Authors:** Yuliya V. Karpievitch, Elizabeth G. Hill, Anthony P. Leclerc, Alan R. Dabney, Jonas S. Almeida

**Affiliations:** 1 Department of Statistics, Texas A&M University, College Station, Texas, United States of America; 2 Division of Biostatistics and Epidemiology, Department of Medicine, Medical University of South Carolina, Charleston, South Carolina, United States of America; 3 Department of Computer Science, College of Charleston, Charleston, South Carolina, United States of America; 4 Department of Bioinformatics and Computational Biology, The University of Texas, M. D. Anderson Cancer Center, Houston, Texas, United States of America; University of East Piedmont, Italy

## Abstract

Many mass spectrometry-based studies, as well as other biological experiments produce cluster-correlated data. Failure to account for correlation among observations may result in a classification algorithm overfitting the training data and producing overoptimistic estimated error rates and may make subsequent classifications unreliable. Current common practice for dealing with replicated data is to average each subject replicate sample set, reducing the dataset size and incurring loss of information. In this manuscript we compare three approaches to dealing with cluster-correlated data: unmodified Breiman's Random Forest (URF), forest grown using subject-level averages (SLA), and RF++ with subject-level bootstrapping (SLB). RF++, a novel Random Forest-based algorithm implemented in C++, handles cluster-correlated data through a modification of the original resampling algorithm and accommodates subject-level classification. Subject-level bootstrapping is an alternative sampling method that obviates the need to average or otherwise reduce each set of replicates to a single independent sample. Our experiments show nearly identical median classification and variable selection accuracy for SLB forests and URF forests when applied to both simulated and real datasets. However, the run-time estimated error rate was severely underestimated for URF forests. Predictably, SLA forests were found to be more severely affected by the reduction in sample size which led to poorer classification and variable selection accuracy. Perhaps most importantly our results suggest that it is reasonable to utilize URF for the analysis of cluster-correlated data. Two caveats should be noted: first, correct classification error rates must be obtained using a separate test dataset, and second, an additional post-processing step is required to obtain subject-level classifications. RF++ is shown to be an effective alternative for classifying both clustered and non-clustered data. Source code and stand-alone compiled versions of command-line and easy-to-use graphical user interface (GUI) versions of RF++ for Windows and Linux as well as a user manual (Supplementary File S2) are available for download at: http://sourceforge.org/projects/rfpp/ under the GNU public license.

## Introduction

Our research was motivated by an analysis of matrix-assisted laser desorption/ionization (MALDI) time of flight (TOF) data. MALDI-TOF data are high dimensional data, characterized by a large number of variables, a (typically) small number of subjects, and a high level of noise. These features complicate subsequent data analysis. Nonetheless, analyses of ion TOF data, including both MALDI- and surface-enhanced laser desorption/ionization (SELDI) TOF data, are used to discover disease-related biomarkers and identify features that discriminate between disease states [1]–[12].

Due to heterogeneous crystallization of the sample/matrix mixture spotted onto MALDI plates, and/or to account for day-to-day instrument variation for both MALDI and SELDI, it is common practice to obtain replicate spectra from the same subject sample, resulting in non-independent (cluster-correlated) subject-level data [13]. Here cluster refers to the collection of samples collected from the same subject. Since multiple samples are collected for the same subject, in principal the samples should be identical. The imperfections in technology and sample processing introduce some variation, resulting in non-identical replicate samples that are more similar to one another than samples from different subjects; that is to say, there is positive correlation between technical replicates from the same subject.

For replicate subject-level observations, we expect the intra-cluster correlation (ICC) to be moderate to high, while for other types of clustered data, the ICC can be quite low. When discriminating between the disease groups, correlated replicate data may not be considered independent [14], [15]. Within-cluster data dependence limits the use of classifiers such as Random Forest (RF) without first altering the data to induce independence, for example, averaging the observations obtained from technical replicates from the same subject [16].

RF is an ensemble of decision trees. Decision trees have been used in bladder cancer diagnosis based on SELDI spectrum protein profiles [11]. Decision trees are examples of weak learners, that is, classifiers characterized by low bias but high variability [16], [17]. Another advantage of decision trees is the ease in which variables and their associated values can be interpreted.

Minor data alterations can result in large changes in the structure of a single tree. RF overcomes this problem of overfitting by averaging across different decision trees. Specifically, each tree is built on a bootstrap sample of the training dataset, so that the bootstrap sample contains, on average, 63% of the unique original samples [16], [18], [19]. Bootstrap sampling, also called bagging (from *bagged aggregation*), exposes the tree construction algorithm to a slightly different subset of the training data for each tree, resulting in a collection of different trees. Since forests typically consist of thousands of trees, the examination of an individual tree or even a select subset of trees is dubious in regards to the effective determination of important variables and corresponding values. For this reason, several variable importance measures have been proposed that rank important variables by considering all trees in the RF [16], [20]. We discuss one of these measures used in RF++ in the Methods section.

A small subsample of variables (the *mtry* parameter in the RF literature) is used at each tree node split, inducing further variation among trees. Together, bagging and variable subsampling reduce overfitting and make RF a more stable classifier than a single decision tree [21], [22]. RFs have been shown to perform comparably to other classification algorithms with respect to both prediction accuracy and the capacity to accommodate large numbers of predictor variables [23]–[25].

RFs have been used in numerous biological applications, including the identification of cancer biomarkers, using a single observation per subject [23], [26], [27]. Vlahou et al. and Svetnik et al. used decision trees and RF, respectively, on averaged replicate data [11], [24]. Although averaging induces independence, a consequence of the resulting data reduction is a loss of information. Moreover, if the number of replicates differs across subjects, averaging masks this imbalance and leads to each subject contributing equally to the resulting classifier.

In our novel RF implementation, we utilize subject-level bootstrapping (described in the Methods section), which enables the effective use of all data samples and allows for unequal contribution from the subjects. In the sections that follow, we describe a generalized Random Forest classifier, RF++, and simultaneously compare it with classical RF approaches for dealing with replicate data. In addition to providing a classification algorithm and measures of variable importance, RF++ accommodates cluster–correlated data in a manner that is consistent with the data's structure.

## Results

### MALDI-TOF Simulated Data

We first investigated the ability of RF++ to correctly identify discriminating variables and classify subjects under conditions of varying: intra-cluster correlation (ICC), numbers of subjects, and numbers of replicates per subject. We grew forests using 125 simulated training datasets with 3 equally discriminating variables as described in the Methods section. We then assessed the forests' classification accuracy and variable selection ability using 25 new simulated testing datasets. We repeated the simulation 200 times to produce stable estimates of the median, 5^th^ and 95^th^ percentiles for the measurements presented below. The simulation study was designed to resemble characteristics observed in the MALDI-TOF data discussed in the previous section.

**Figure 1:**
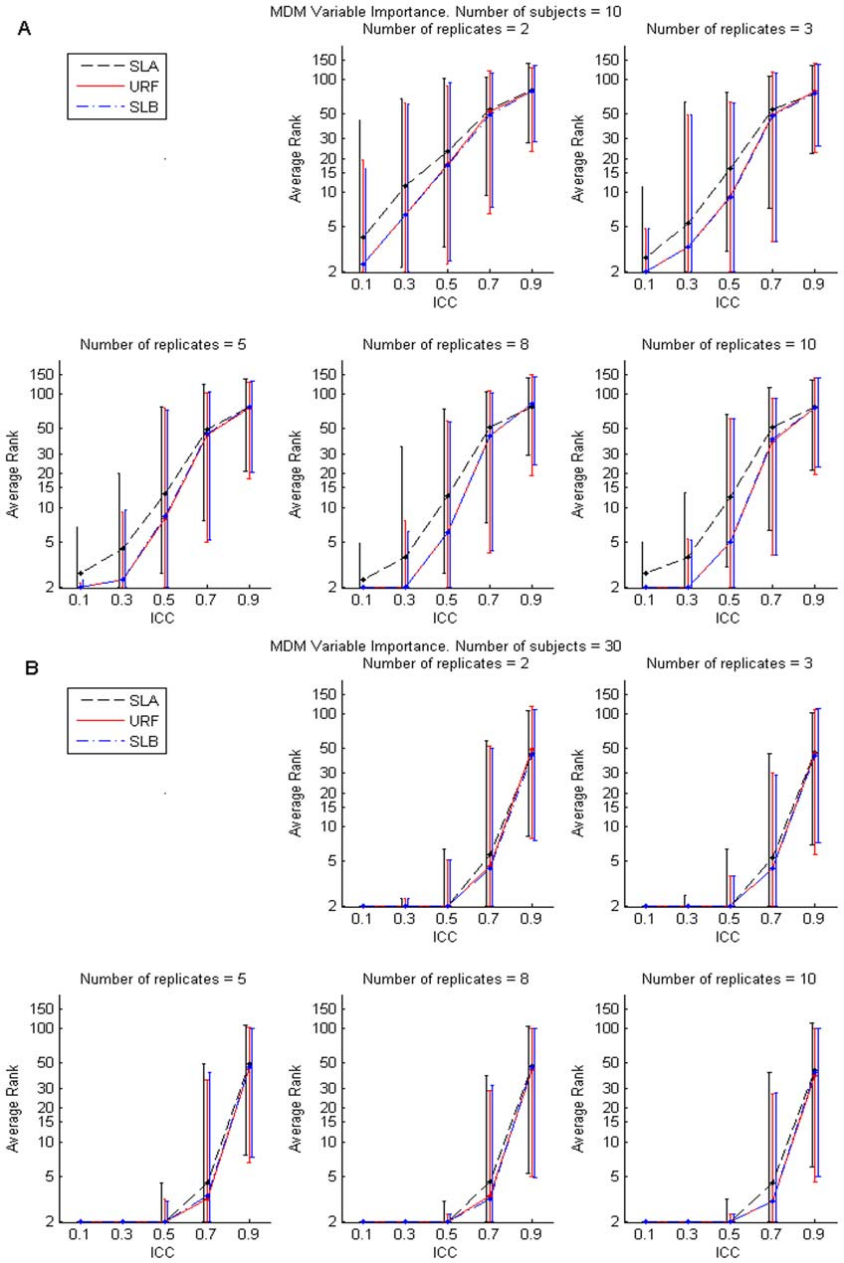
Mean Decrease in Margin (MDM) variable importance. Median and the 5^th^ and 95^th^ percentiles of the logarithm of the average ranks of the MDM variable importance scores for the three discriminating variables for 200 simulations (see text) with forests grown on ten subjects (panel A) and 30 subjects (panel B). The median is depicted by a dot, and vertical bars represent the 5^th^ and 95^th^ percentiles. Vertical bars are separated artificially along the x-axis to improve visual representation. Results for SLB and URF forests grown on subject-level bootstrapped data correspond to dot-dashed blue and solid red lines, respectively. The dashed black lines correspond to SLA.

**Figure 2:**
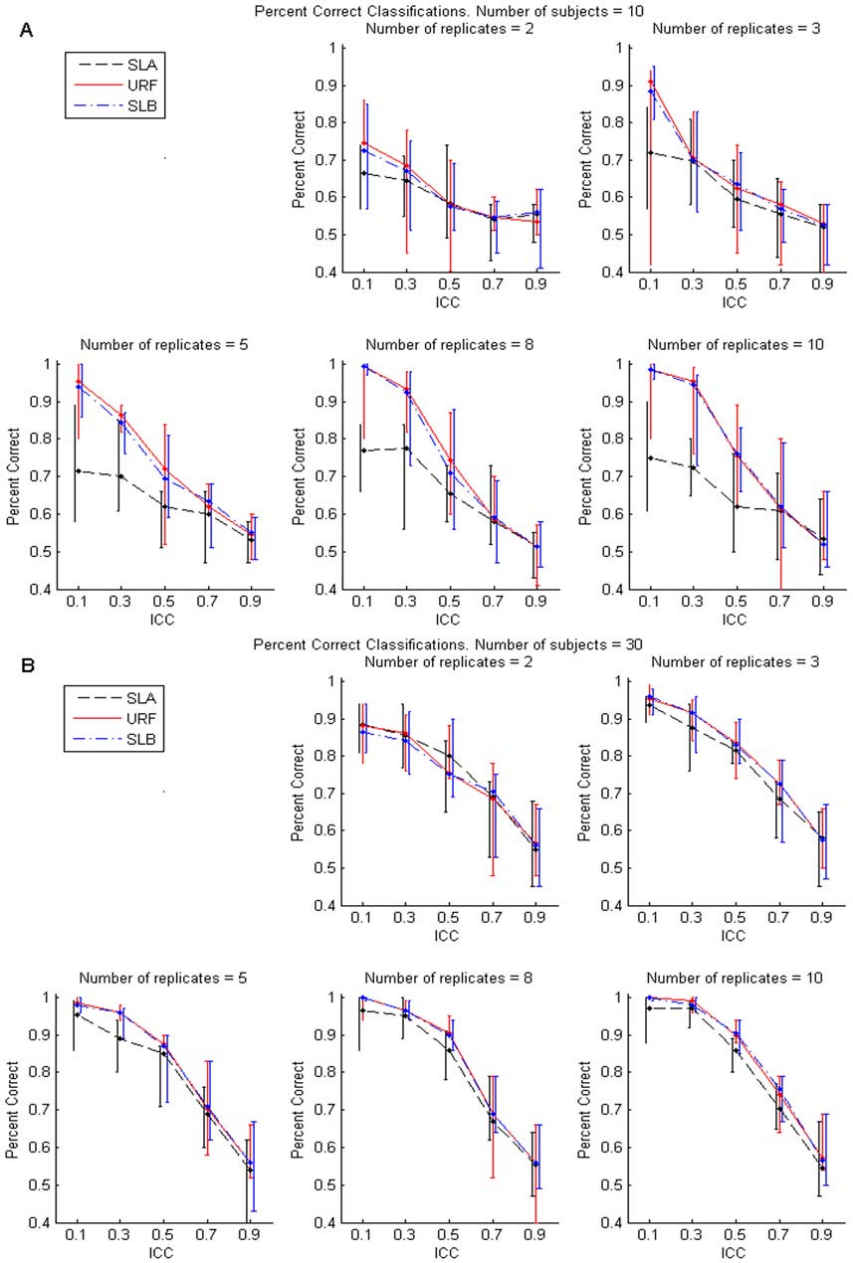
Proportion correct classification. Median and the 5th and 95th percentiles of the proportion correct subject-level classification for 600 simulations (see text) with forests grown on ten subjects (panel A) and 30 subjects (panel B). The median is depicted by a dot, and vertical bars represent the 5th and 95th percentiles. Vertical bars are separated artificially along the x-axis to improve visual representation. Results for SLB and URF forests grown on subject-level bootstrapped data correspond to dot-dashed blue and solid red lines, respectively. The dashed black lines correspond to SLA.

**Figure 3:**
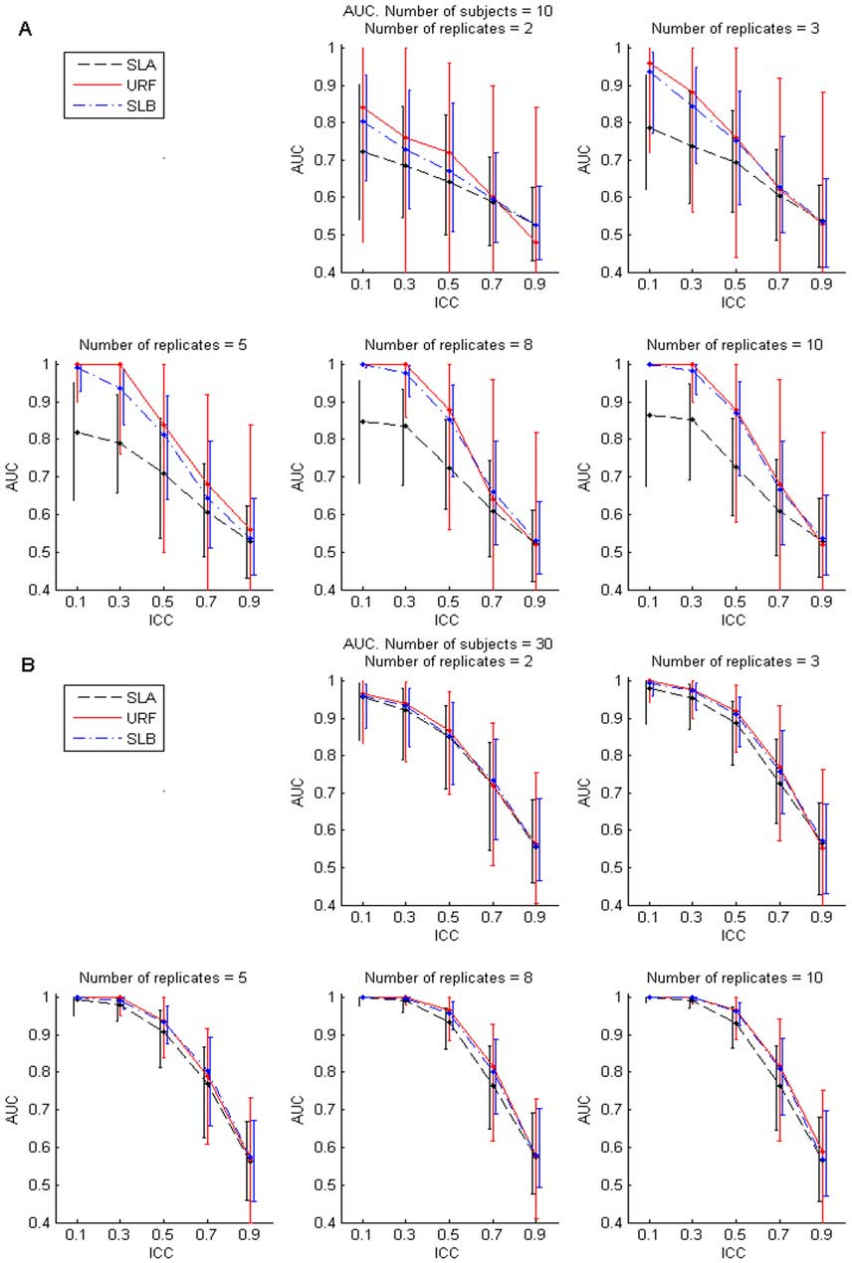
Area under the receiver operating characteristic curve (AUC). Median and the 5th and 95th percentiles of the AUC for 200 simulations (see text) with forests grown on ten subjects (panel A) and 30 subjects (panel B). The median is depicted by a dot, and vertical bars represent the 5th and 95th percentiles. Vertical bars are separated artificially along the x-axis to improve visual representation. Results for SLB and URF forests grown on subject-level bootstrapped data correspond to dot-dashed blue and solid red lines, respectively. The dashed black lines correspond to SLA.

Figures 1, 2, and 3, depict results corresponding to forests grown by RF++ with subject-level bootstrap sampling (SLB), dot-dashed blue lines; results corresponding to forests grown assuming all samples are i.i.d. (URF), solid red lines; and results corresponding to forests grown on subject-level averaged (SLA) samples, dashed black lines. For each performance measure, we present results only for ten (five in each class) and 30 (15 in each class) subjects. Simulation results for 20, 50 and 100 subjects were qualitatively similar to those shown for 30 subjects, and were therefore excluded in the interest of brevity.

#### Variable Importance

To compare each method's ability to select discriminating variables, we ranked the variable importance scores produced by the simulations for each forest and computed an average rank for the 3 equally discriminating variables. The best possible average rank was 2 when all discriminating variables were in the top 3 positions. Figure 1 shows the median and the 5^th^ and 95^th^ percentiles of the logarithm of the average rank for the 3 discriminating variables of the 200 simulations for the SLB, URF and SLA forests. Results are shown for simulations with 10 and 30 subjects in Figures 1A and 1B, respectively.

The RF++ variable importance ranks obtained from SLB and URF forests were consistently lower than the ranks from SLA forests. The ability to select discriminating variables decreased for both SLB and SLA forests as the ICC increased. This is expected, since the effective sample size for clustered data is 
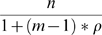
 which approaches the sample size for the SLA method when 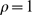, 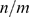. Here *n* is total number of samples, *m* is number of samples within a subject (cluster), and *ρ* is intracluster correlation coefficient. For 20 subjects or more, the intervals defined by the 5^th^ and 95^th^ percentiles of the average rank distribution for the three discriminating variables were uniformly lower and narrower for SLB and URF forests than for SLA forests.

For 10 subjects, SLB and URF forests performed better for all but the highest value of ICC = 0.9. There was little difference in the accuracy of variable selection between the SLB and URF forests, suggesting that both bootstrap methods can be equally used for variable selection.

As the number of subjects increased, all of the forests identified the discriminating variables with increasing accuracy across a wider range of ICC values. Note, for example, the straight line in Figure 1B at average rank = 2 for the ICC values from 0.1 to 0.5, which indicates nearly perfect identification of the 3 discriminating variables in this ICC range. The average rank increased to 4 or greater for ICC = 0.7 with a large increase in the width of the interval defined by the 5^th^ and 95^th^ percentiles. Figure 1 demonstrates that SLB and URF forests identify important variables equally well and usually better then SLA forests. Specifically, SLB and URF forests in our simulations produced lower discriminating variable importance ranks than the SLA forests for ICC values between 0.1 and 0.7. All forests performed poorly at ICC = 0.9 with median average ranks above 76.

### Classification Accuracy

#### Proportion Correctly Classified

Because RF++ is constructed to accommodate clustered data, it summarizes classification both at the replicate and subject level. Replicates are classified based on the majority vote of all trees in the forest. Subjects are then classified by majority vote of their replicates, as described in the Methods section.

Figures 2A and 2B show the median and the 5^th^ and 95^th^ percentiles of the proportion of subjects correctly classified for SLA, SLB and URF forests across 200 simulated test data sets for ten and 30 subjects, respectively. As expected, the algorithm predicted class membership with decreasing accuracy as the ICC increased, but the classification accuracy of SLA forests was uniformly equal to or less than that of SLB and URF forests (except for a single case for 30 subjects with 2 replicates and ICC = 0.5). This is most notable for small numbers of subjects (Figure 2A), with a nearly 15% difference in accuracy for the small values of ICC. The differences between the forests decreased as ICC increased, due to effective sample size for SLB and URF forest approaching the sample size of SLA forest as explained above. We also note that the forests achieved similar classification performance as the number of subjects increased. We observed no difference in classification performance between SLB and URF forests.

#### Area Under the Receiver Operating Characteristic Curve

To assess classification performance of the forests in a manner independent from the decision threshold (for majority vote the decision threshold is 0.5, i.e. above 50% trees to vote for a particular classification in a two class classification), we computed the area under the receiver operating characteristic curve (AUC) [28]. Figures 3A and 3B show the median and the 5^th^ and 95^th^ percentiles of AUC for the three forests across 200 simulations for 10 and 30 subjects, respectively. URF forests produced greater median AUCs for 10 subjects with SLB tracing closely and SLA performing up to 18% worse. Although URF and SLB forests had similar median AUCs, SLB forests yielded consistently narrower 90% credible intervals than URF forests, representative of a more stable performance. Differences in AUCs among all forests decreased for 50 subjects and were negligible for 100 subjects. All forests produced similar 90% credible intervals for 100 subjects. It is noteworthy that all forests had similar performance at the extreme ICC values of 0.1 and 0.9 for numbers of subjects 30 or larger (Figure 3B), but URF and SLB forests had greater AUCs than SLA forests at intermediate ICC values (0.3, 0.5, 0.7).

### Application to Esophageal Cancer Data

We analyzed MALDI-TOF spectra derived from serum samples of esophageal cancer patients to further validate the results in classification accuracy on real MS data. Sera were obtained from 38 (30 cancer and 8 control) subjects, fractionated, and analyzed by MALDI-TOF MS. We obtained 507 spectra with the following numbers of replicates per subject: 28 subjects had 12 replicates; 5 subjects had 24 replicates; 4 subjects had 11 replicates; and 1 subject had only 7 replicates. Spectra were preprocessed using PrepMS with the mean spectrum smoothing threshold set to 20, individual spectra smoothing threshold set to 16, and signal-to-noise ratio set to 20 [29]. Intensities below 2000 kDa were considered matrix noise and were eliminated from the analysis. A total of 185 peaks were identified. Spectra were further normalized with EigenMS to eliminate any systematic bias [30]. One significant eigenpeptide (trend) that explained 88.25% of the variation was detected and its effects were removed.

We grew URF, SLB and SLA forests each with 2001 trees. We performed 100 experiments dividing the subjects into training and testing datasets. Two-thirds of the 38 subjects (26 subjects) were used for training, randomly choosing 6 of the subjects from the control group and 20 of the subjects from the disease group, respectively. The remaining 12 subjects were used for testing.

**Figure 4:**
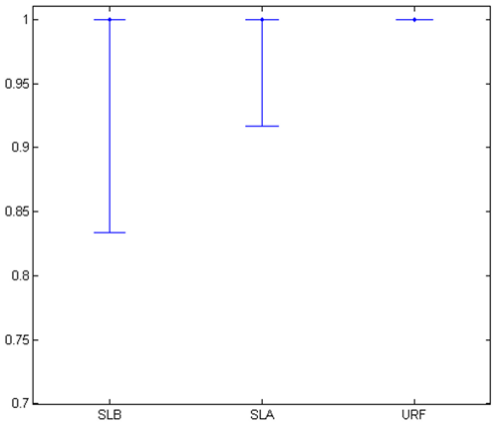
Percent correct classifications in esophageal data. Dots represent the median percent correct classifications and whiskers represent 5th and 95th percentiles.

As depicted in Figure 4, all three forests performed similarly with 50th and 95th percentiles at 100% correct classification. Fifth percentiles differed with 83% for SLB, 91% for SLA and 100% for URF. These results are otherwise consistent with the results obtained using simulated data.

## Discussion

Our motivation for this research was biomarker discovery based on MALDI-TOF mass spectrometry (MS) data. MS data are characterized by both a small number of subjects and a large number of variables (most of which are non-discriminating between the classes), and require the use of robust classifiers that can handle such constraints. Previously it was unclear whether correlation among replicate spectra (common with data obtained in MS experiments) should be specially handled.

Our study results indicate that RF++ provides an approach to the analysis of cluster-correlated data that matches the performance of the existing (unmodified) RF algorithm applied at the sample level. The only caveat is that OOB error rate produced by the URF forests is typically an underestimate. Error rates for clustered data analyzed with URF should properly be estimated on a separate test dataset. We further demonstrated that the performance of SLB forests is typically better than the performance of SLA forests with respect to the detection of discriminating variables, classification accuracy, and AUC.

When the ICC was near zero, we observed substantial gains in variable selection and classification capabilities for both URF and SLB as compared to SLA forests. This is not surprising because the replicates are nearly independent when the ICC is small, and therefore averaging results in the greatest loss of information. Conversely, when the ICC is large (close to 1), the within-subject data are nearly identical and there is little additional information in the replicates. Subsequently, we observed little performance improvement when comparing forests as ICC approaches 1. Overall, for number of subjects greater then 100 any of the three forests discussed here will produce similar prediction and variable selection accuracy.

Although this manuscript has focused on the analysis of technical replicates, dependence must also be taken into account in longitudinal studies and designs in which the class assignments associated with subject replicates are potentially different. Our approach can be extended to longitudinal data by the utilization of a modified impurity measure [31]–[33] and to address the issue of the correlated predictor variables [34].

This report mainly considers the issue of classification of data clustered at the subject level. Some of the functionality of (the original) Breiman's RF has been omitted, such as regression analysis where the outcomes are continuous and weighted class analysis for unbalanced data sets. Missing values imputation for MS-based proteomics data has been described in Karpievitch et al. and can be performed prior to classification. We consider these features important and plan to incorporate them into future RF++ implementations.

MS data are an example of data with a small number of subjects and a large number of variables. The use of subject level bootstrapping (SLB) by RF++ is shown to be advantageous for the analysis of such data, because the sampling scheme is designed to accommodate data with multiple measurements for a given subject (e.g. technical replicates). Perhaps surprisingly, our results also suggest that it is still reasonable to utilize URF for the analysis of cluster-correlated data with two caveats: first, correct classification error rates must be obtained using a separate test dataset, and second, an additional post-processing step is required to obtain subject-level classification. Our studies also show that, even for moderate values of ICC, forests grown utilizing all available data (SLB or URF) classify and identify discriminating variables with greater accuracy than forests grown on averaged samples.

RF++ constitutes a useful research tool contribution providing an easy-to-use graphical interface and eliminating the manual reconfiguration and recompilation requirements of Breiman's existing FORTRAN version. The SLB additions to the RF algorithm implemented in RF++ are valuable to researchers analyzing cluster-correlated data. RF++ can be used to effectively analyze both clustered and non-clustered data.

## Methods

### RF++ algorithm

RF++ is a classifier capable of analyzing cluster-correlated data. It was developed as a C++ implementation of the RF algorithm, as described by Breiman [16], with additional functionality specific to the structure of cluster-correlated data.

First, RF++ grows each tree on a bootstrap sample (a random sample selected with replacement) at the subject-level rather than at the replicate-level of the training data. Individual trees are unpruned classification/decision trees grown using the *Gini* impurity score. A particular subject is chosen at random from the pool of all available subjects and all of its replicates are allocated to the in-bag dataset. As mentioned previously, approximately 63% of the individual samples are in-bag (IB) and the remainder are held out in order to compute a runtime error estimate on the out-of-bag (OOB) samples. When using subject-level bootstrapping we also expect about 63% of the *subjects* to be placed in-bag. Subject-level bootstrapping ensures that bootstrap samples are constructed from independent units, or in this case, subjects, with correlated replicates collected from those subjects. Subject-level bootstrapping overcomes the problem of potentially exposing individual trees to all subjects (See Supplementary File S1 Section 1).

Since our primary goal is to provide a classification method applicable to cluster-correlated data, we are only interested in estimating the classification error rate and not in performing inference on the model components. For these reasons it is not necessary to include covariance estimates in the tree construction. Using the subject-level bootstrap results in unbiased classification error rate estimation, regardless of whether the dependence within clusters is incorporated into the tree construction.

**Figure 5:**
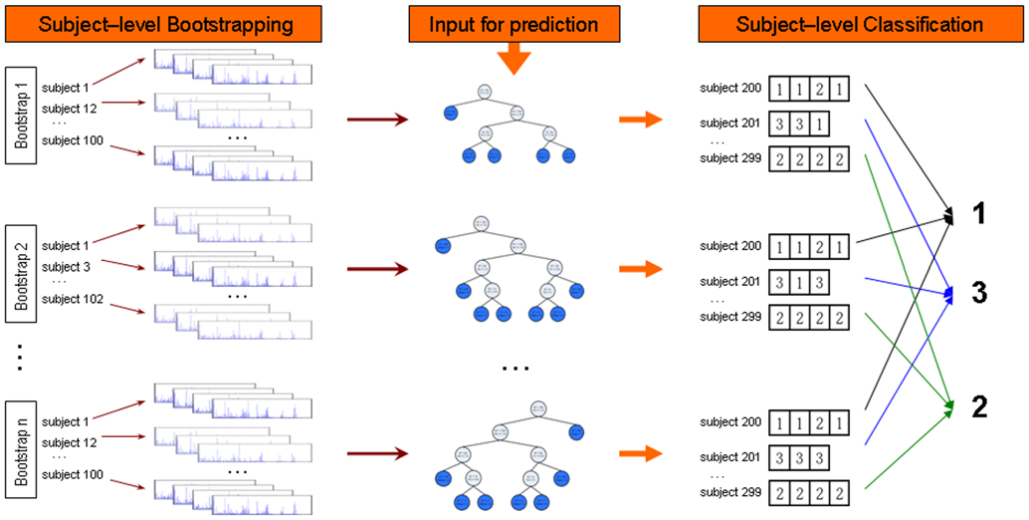
RF++ outline. Each tree is grown on a different subject-level bootstrap set of samples (left) producing a forest (middle). New subject samples are piped down the forest and each tree casts a vote for each sample. A subject classification is computed as the class with the maximum number of votes across all samples for that subject among all trees (right). Proportions of votes for each class are also produced.

Second, we provide a means for computing subject-level classification. Specifically, we first classify subject replicates at the sample-level and then perform a majority vote across the subject replicates in order to compute subject classification. The ability to classify at the subject level in addition to the replicate level is useful when analyzing clustered data in which all subject replicates belong to the same class. In such cases we are ultimately interested in subject-level classification, and not just classification of individual replicates from the same subject. Figure 5 illustrates RF++ replicate- and subject-level classification. If different replicates for the same subject belong to different classes (such as measurements taken at different time points), only replicate-level classification is produced.

Third, like Breiman's original Random Forest, RF++ provides an error rate based on OOB data [16]. The OOB replicate error rate estimate is always computed. When all subject replicates belong to the same class, we compute an unbiased running OOB subject-level error estimate. Occasional misclassifications (e.g. one or two replicate misclassifications out of a collection of replicates) generally have little effect on the final forest subject-level error rate.

It is important to note that even when subject-level error rate and classifications are computed, the replicate-level error rate and classifications are still computed and made available for closer examination on an individual replicate level. For example it may be of interest to know that 5 out of 10 subject replicates are correctly classified (replicate-level error rate of 50%). RF++ also produces proportions of votes for each class which gives an estimate of the probability that the subject (and/or the replicate) falls within a particular class. These proportions can be used in decision making models that use different cut-off values to distinguish between classes. For example, in a two class problem with 0/1 outcomes in which the cut-off is 0.5. However, one might want to explore the predictive performance (e.g. sensitivity, specificity, AUC) over a range of thresholds, and this is facilitated by the reporting of estimated probabilities of class membership.

#### RF++ Variable Importance Measures

RF++ utilizes permutation-based variable importance measure implemented in Breiman's original RF. It has been shown that other variable importance measures (such as *number-of-times-used* and *Gini importance*) do not perform as well with respect to detecting discriminating variables [20]. *Number-of-times-used*, a count of how many times a variable is used to split a node in a forest, is susceptible to random variable subsampling effects at each node split. This means that, due to the selection of a variable from a much smaller set (usually a subset of size 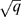, where *q* is the total number of variables in the data set), the variable may be chosen for a split even if it is not truly discriminating. In fact, *number-of-times-used* is not implemented in the current FORTRAN version of RF. The *Gini importance* measure, on the other hand, is more robust [35]. It quantifies the decrease in the “*Gini impurity score*” computed at each node split, and can be accumulated for each variable across all trees. *Gini importance* has been shown to be biased towards variables with larger numbers of possible values, including continuous variables [20]. For example, Gini importance ranks a continuous variable as more importance than a binary variable even if both are equally discriminating.

The permutation-based variable importance measure is the least biased towards variables with a large range of values, as described by Strobl et al. 2007. Systems biology studies produce variables with wide continuous ranges, and thus we are less likely to encounter bias when using a permutation-based variable importance measure. RF++ provides two variations of the permutation-based importance measure. In RF++ the simple permutation-based importance measure for variable *v*, 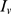, is described in Equation 2 as
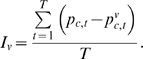
(2)


Here 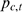 is the proportion of correctly classified replicates out of the total number of OOB replicates in a given tree *t*, 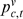 is the proportion of OOB replicates correctly classified after variable *v* has been randomly permuted across all OOB replicates for tree *t*, and *T* is the total number of trees in the forest.

The second variable importance measure included in RF++ is the mean decrease in margin (MDM) for each variable as shown in Equation 3. Margin is defined as the proportion of votes for the correct class minus the largest proportion of votes for an incorrect class (that is, the incorrect class that received the largest number of votes). The mean decrease in margin for variable *v* is defined as
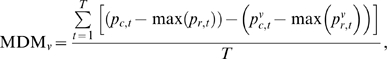
(3)where 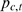 is the proportion of correctly classified replicates out of the total number of OOB replicates for a given tree, *t*, 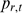 is the proportion of OOB replicates incorrectly classified; 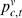 and 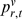 are the proportions of correctly and incorrectly classified OOB replicates, respectively, after variable *v* has been randomly permuted within the OOB replicates for tree *t*; and T is the total number of trees in the forest.

### Training and Testing Data Generation

To test the performance of the RF++ algorithm, we generated training and testing datasets with cluster-correlated observations in which each subject had more than one replicate and where some covariates may also be correlated. Our goal was to simulate data derived from the replicate spectra obtained from MS TOF experiments. Therefore, in our simulations, we considered data with a small number of subjects and a large number of variables, most of which possessed no discriminating information. We modelled data that has already been preprocessed, i.e. aligned along the m/z scale, denoised, baseline corrected and where peaks were detected. As a result the number of peaks are usually reduced from tens of thousands to hundreds and all peaks have the same m/z scale [29], [36], [37]. MS TOF data preprocessing is an essential step that is performed prior to analysis with any classifier including RF++.

Our simulation study addressed the effects of varying ICC on variable selection and classification abilities of RF++. The ICC is defined as the proportion of total variance attributable to between cluster variability, and is given by
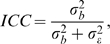
(4)where 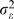 is the within cluster variance and 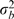 is the between cluster variance, i.e. the variance of the random effects, and as such influences dispersion among the cluster locations. The cluster locations become increasingly ‘spread out’ as 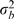 increases. Thus we refer to 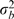 as the ‘between cluster variance’. In our simulations, we fixed 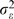 at 1, and, based on Equation (4), selected 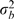 values of 0.11, 0.43, 1, 2.33 and 9 to produce ICC values of 0.1, 0.3, 0.5, 0.7, and 0.9, respectively.

The log-transformed normalized intensities in real MS data are less skewed, with more similar variances and are roughly normally distributed [28], [36]. We therefore simulated all log peak intensities from a normal distribution. For convenience, we chose a mean of 6 and variance of 1. For peaks that were discriminating (randomly selected a priori), we took the original peak mean and added (subtracted) one standard deviation to (from) it producing two distinct disease group means corresponding to the disease and control classes. Standard deviations for the two disease groups were unchanged. For each subject *i* and peak *k*, we generated *j* replicate m/z log peak values using the corresponding means and adding a subject-specific random effect, 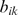, assuming that 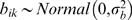. For a given subject, the value of 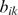 remained constant for all m/z log peak intensity replicates, thereby creating a common ‘shift’ in that subject's observations that corresponded to the specified m/z value. To provide additional variation to the values, we added noise, given by 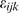, which we assumed followed a standard normal distribution. Additionally, we assumed that the random effects and the errors were independent. Conditional on the random effect, the subject replicates were assumed to be independent, but marginally the within-subject observations were correlated. For a given m/z value, we generated replicate log peak intensities using
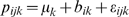
(5)where *i* is the subject index, *j* is the replicate index for subject *i*, *k* is the peak index, and 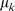 is the mean log peak intensity for the specified m/z value corresponding to the disease group of the *i^th^* subject.

We produced replicate log peak intensities corresponding to 185 total m/z values for each subject. Three of the m/z values (peaks) were discriminating features, and the remaining 182 m/z values were pure noise. Noise peaks were generated from the same distribution as the discriminating peaks but with the means of the two disease groups being equal. For two of the discriminating peaks, we selected 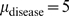 and 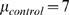. For the remaining discriminating peak, we specified 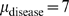 and 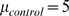.

In the design above, the peaks are uncorrelated. This is not the case in real MS datasets. For this reason, we generated datasets with correlation between peaks. We generated vector 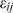 from a multivariate normal distribution 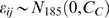, where 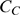 is the correlation matrix computed from the esophageal cancer dataset described in the Results section. Readers interested in a more detailed description of the data generation and the classification and variable selection accuracy of the forests on these data are referred to Section 3 of the Supplementary File S1.

### Simulation study

In our simulation study we compared the impact of varying ICC on variable selection and classification performance for 5 different ICC values (0.1, 0.3, 0.5, 0.7 and 0.9), 5 different numbers of subjects (10, 20, 30, 50 and 100), and 5 different numbers of replicates within subjects (2, 3, 5, 8 and 10). We therefore generated 125 training data sets to accommodate all possible combinations of the 3 parameters. In the training data, the total number of subjects was always equally divided between 2 classes. Thus, a training data set with 10 subjects had five disease and five control subjects.

To test the prediction and variable selection accuracy of RF++ we fixed the number of subjects to 100 and generated 25 test data sets. We again allocated equal numbers of subjects to each class to facilitate easy comparison.

To mitigate the effects attributable to random number generations for each data set, and to provide measures of uncertainty in our estimates, we repeated each simulation 200 times for each combination of ICC, number of subjects, and number of replicates. For each simulation, we obtained the average importance ranks of the three discriminating variables based on the MDM variable importance scores, the proportion of subjects correctly classified, and the AUC. Based on the empirical distributions of these performance measures, we summarized our results by reporting the median and the 5^th^ and 95^th^ percentiles.

In each of these 200 simulations we regenerated both the training and testing data sets. For each of the training data sets we grew 3 different types of forests: a SLA forest grown on averaged subject samples, a SLB forest, and an unmodified Breiman's forest, URF. All forests contained 2001 trees. We subsequently tested each forest's performance using the same testing data set. For testing of the SLA forest, subject replicates were averaged.

## Supporting Information

File S1 Supplementary materials
(0.11 MB DOC)

File S2 RF++ User Manual
(0.34 MB PDF)
