# Supplementary material for: An Introspective Comparison of Random Forest-Based Classifiers for the Analysis of Cluster-Correlated Data by Way of RF++
Source: PLoS One. 2009 Sep 18;4(9):e7087. doi: 10.1371/journal.pone.0007087 (PMC2739274; doi:10.1371/journal.pone.0007087)
Supplement: File S1 — Supplementary materials (0.11 MB DOC) [file pone.0007087.s001.doc]

Supplementary material to

## *RF++: Generalized Random Forest-based classifier for cluster-correlated data*

Yuliya V Karpievitch^1,2,3§^, Elizabeth G Hill^2^, Anthony P Leclerc^4^, Alan R Dabney^3^, and Jonas S Almeida^1^

# Section 1 – Subject-level vs. sample-level bootstrapping.

When analyzing replicate data, bootstrapping at subject level is necessary because resampling at replicate level will most likely expose each individual tree to all subjects. Bootstrap assures that approximately 63% of all replicates will end up in the in-bag training set for a particular tree, thus the tree will be unaware of the rest of the independent samples in the dataset (OOB samples) and the error estimate will be unbiased.

If the samples are not independent, i.e. subject replicates have higher correlation with themselves then with replicates from other subjects, then exposing a tree to just one of the replicates from a subject will ‘give out’ information about the rest of that subject’s replicates to the tree and increase the correlation between the trees in the forest. This will result in overfitting to the training data, as a tree will most likely be exposed to replicates from all subjects. Table 1 shows empirically determined numbers of samples and subjects (out of 100 subjects) that end up in the in-bag subset of the data on which a tree is grown. The results shown in Table 1 are averaged over 10,000 bootstrap sampling iterations.

| Number of replicates per subject | Percent replicates in-bag | Percent subjects  in-bag |
| --- | --- | --- |
| 2 | 63.34 | 86.65 |
| 5 | 63.28 | 99.35 |
| 10 | 63.44 | 99.98 |

## Table 1 - Percent of samples and subjects in-bag when bootstrapping is performed at replicate/sample level.

As can be seen in Table 1, for 100 subjects with 10 replicates each, 10,000 sample(replicate)-level bootstrap samples contain, as expected, 63% of the individual samples/replicates in the in-bag. Unfortunately sample-level resampling samples at least one replicate from each subject resulting in almost 100% of the subjects having at least one replicate in-bag for each tree. For 100 subjects with only 2 replicates for each, 10,000 sample-level bootstrap samples contain, as expected, 63% of the individual samples/replicates and 86.65% of the subjects in-bag.

The number of subjects that are placed into the in-bag samples are affected by the number of replicates per subject. A sample from the subject with larger number of replicates has a higher chance of being selected at random. Thus a subject with large number of replicates has higher chance of being placed into the in-bag if the resampling is done at the sample level. Trees grown on subject-clustered data using sample-level resampling will be overfit to the training data, the correlation between trees will increase and the estimated OOB error rate will be unreliable. Generating more trees that are overfit to the training data will produce an overfit forest that will fail to generalize on an independent testing dataset and thus will be inadequate for future predictions.

In RF++ we implement subject-level bootstrapping which samples from the pool of independent units (subjects). Individual trees are thus exposed to about 63% of all the available subjects and are not overfit to the data.

# Section 2 – Variable Importance Measurements

RF++ provides two variable importance measures. As described in the manuscript both are permutation-based. In our simulations both measures produced similar results, and for that reason we show the results of variable selection using only the MDM importance measure in the manuscript. Both measures have similar accuracy, thus the user is free to decide which measure to use or look for agreement in the measures.

During the development of RF++ a second variable importance measure was useful to validate the calculation and accuracy of the MDM measure. We have retained this second importance measure simply to provide an alternative.

# Section 3 – Analysis of Simulated Data with Correlated Peaks.

The log-transformed normalized intensities in MS data are less skewed, with more similar variances and are roughly normally distributed. We therefore simulated all log peak intensities from a normal distribution. For convenience, we chose a mean of 6 and variance of 1. For peaks that were discriminating (randomly selected a priori), we took the original peak mean and added (subtracted) one standard deviation to (from) it producing two distinct disease group means corresponding to the disease and control classes. Standard deviations for the two disease groups were unchanged. For each subject *i* we generated *j* replicate m/z log peak values using the corresponding means and adding a subject-specific random effect, $b_{ik}$, assuming that $b_{ik}$ $\sim\text{Normal}(0,\sigma_{b}^{2})$. For a given subject, the value of $b_{ik}$ remained constant for all m/z log peak intensity replicates, thereby creating a common ‘shift’ in that subject’s observations that corresponded to the specified m/z value. To provide additional variation to the values, we added noise, given by$\varepsilon_{ijk}$, which we assumed followed a standard normal distribution, $\varepsilon_{ijk}\sim N(0,1)$. Additionally, we assumed that the random effects and the errors were independent. Conditional on the random effect, the subject replicates were assumed to be independent, but marginally the within-subject observations were correlated. For a given m/z value, we used the following model for replicate log peak intensities:

$$p_{ijk} =\mu_{k}+ b_{ik}+\varepsilon_{ijk},$$

where *i* is the subject index, *j* is the replicate index for subject *i*, and *k* is the peak index. Thus,

$$Cov(p_{ijk},p_{ij^{*}k})=\sigma_{b}^{2}$$

$Cov(p_{ijk},p_{ijk^{*}})=0$ (2)

$Var(p_{ijk})=\sigma_{b}^{2}+ 1$,

for any $j^{*}\neq j$ and $k^{*}\neq k$.

We produced replicate log peak intensities corresponding to 185 total m/z values for each subject. Three of the m/z values (peaks) were discriminating features, and the remaining 182 m/z values were pure noise. Noise peaks were generated from the same distribution as the discriminating peaks but with the means of the two disease groups being equal. For two of the discriminating peaks, we selected $\mu_{\text{disease}}=5$ and$\mu_{\text{control}}=7$. For the remaining discriminating peak, we specified $\mu_{\text{disease}}=7$ and$\mu_{\text{control}}=5$.

Because peaks in MS data are correlated, we generated data where we imposed correlation between the peaks. We did so by obtaining an estimated correlation matrix,$C_{c}$, from the esophageal cancer dataset described in the manuscript. The data set was preprocessed using PrepMS (spectra were denoised, baseline corrected, normalized, and peaks were identified) which resulted in the identification of 185 peaks [1, 2].

We impose correlation by generating vector$\varepsilon$ from a multivariate normal distribution with covariance matrix$C_{c}$ equal to the estimated correlation matrix from esophageal cancer dataset, $\varepsilon_{ij}\sim\mathop{N}\limits_{185} (0,C_{c})$. The ones on the diagonal of the $C_{c}$ allow us not to alter the variance of the peaks. On the other hand, the non-zero values on the off-diagonal of the matrix allow us to impose correlation between different peaks. Everything else was left unchanged.

Given this design, we have:

$$Cov(p_{ijk},p_{ij^{*}k})=\sigma_{b}^{2}$$

$Cov(p_{ijk},p_{ijk^{*}})=\left[C_{c}\right]_{kk^{*}}$ (3)

$Var(p_{ijk})=\sigma_{b}^{2}+ 1$,

for any $j^{*}\neq j$ and $k^{*}\neq k$, where $\left[C_{c}\right]_{kk^{*}}$ is the element in the $k^{th}$ row and column of $C_{c}$. The error terms thus have exactly the same correlation structure as the cancer data. The actual peak measurements will have slightly lower correlation, since $Corr(p_{ijk},p_{ijk^{*}})=\left[C_{c}\right]_{kk^{*}}/(\sigma_{b}^{2}+1)$. However, the simulation setup should allow for an adequate assessment of a plausible correlation structure between peaks.

Figure 1 shows visible difference in trends of the peaks for two spectra from the same subject with uncorrelated peaks and correlated peaks.


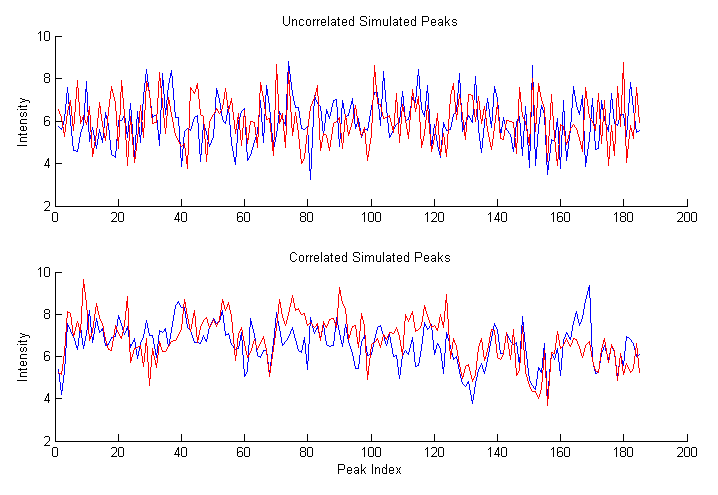


## Figure 1 - Uncorrelated vs. correlated peaks

Two spectra with uncorrelated peaks that come form the same subject (top) and two spectra from the subject with correlated peaks (bottom).

References

1. Karpievitch YV, Hill EG, Smolka AJ, Morris JS, Coombes KR, Baggerly KA, Almeida JS: **PrepMS: TOF MS data graphical preprocessing tool.** *Bioinformatics* 2007, **23:**264-265.

2. Morris JS, Coombes KR, Koomen J, Baggerly KA, Kobayashi R: **Feature extraction and quantification for mass spectrometry in biomedical applications using the mean spectrum.** *Bioinformatics* 2005, **21:**1764-1775.
