# Supplementary material for: An Introspective Comparison of Random Forest-Based Classifiers for the Analysis of Cluster-Correlated Data by Way of RF++
Source: PLoS One. 2009 Sep 18;4(9):e7087. doi: 10.1371/journal.pone.0007087 (PMC2739274; doi:10.1371/journal.pone.0007087)
Supplement: File S2 — RF++ User Manual (0.34 MB PDF) [file pone.0007087.s002.pdf]

RF++
User Manual
Version 1.0

Home Pages: http://sourceforge.net/projects/rfpp

Contact:

Yuliya Karpievitch: **yuliya@stat.tamu.edu**

Anthony Leclerc: **leclerc@cs.cofc.edu**

Table of Contents

Training (growing) a new Forest 3

Training Data Information 3

Forest Parameters 4

Training Results 5

Testing a Forest and Making Predictions 6

Saving forest, variable importance scores and classifications 9

Loading an Existing Forest 11

Forest File Description 12

GUI

Training (growing) a new Forest

From the initial RF++ window we can select to train a new forest or open an existing forest.
To train a forest, select the ‘Train’ menu item from the ‘File’ pull down menu. This will open a training data tab.

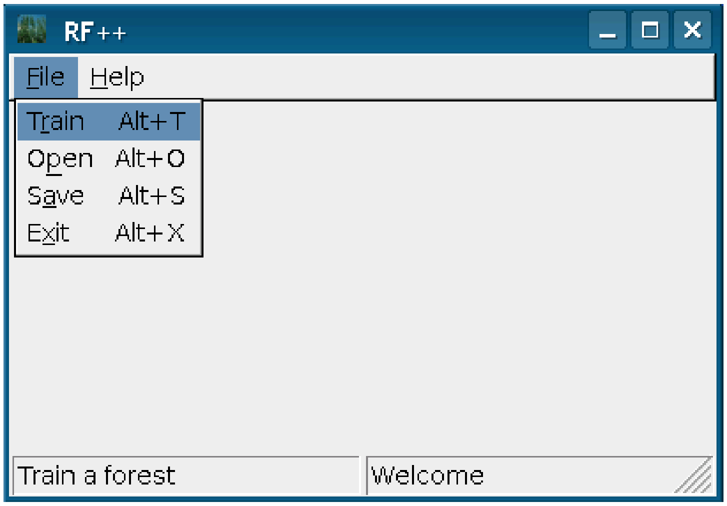

Training Data Information

Two files are provided with the executable: train_100_10_1.txt and test_100_10_1.txt. These are training and testing files respectively.

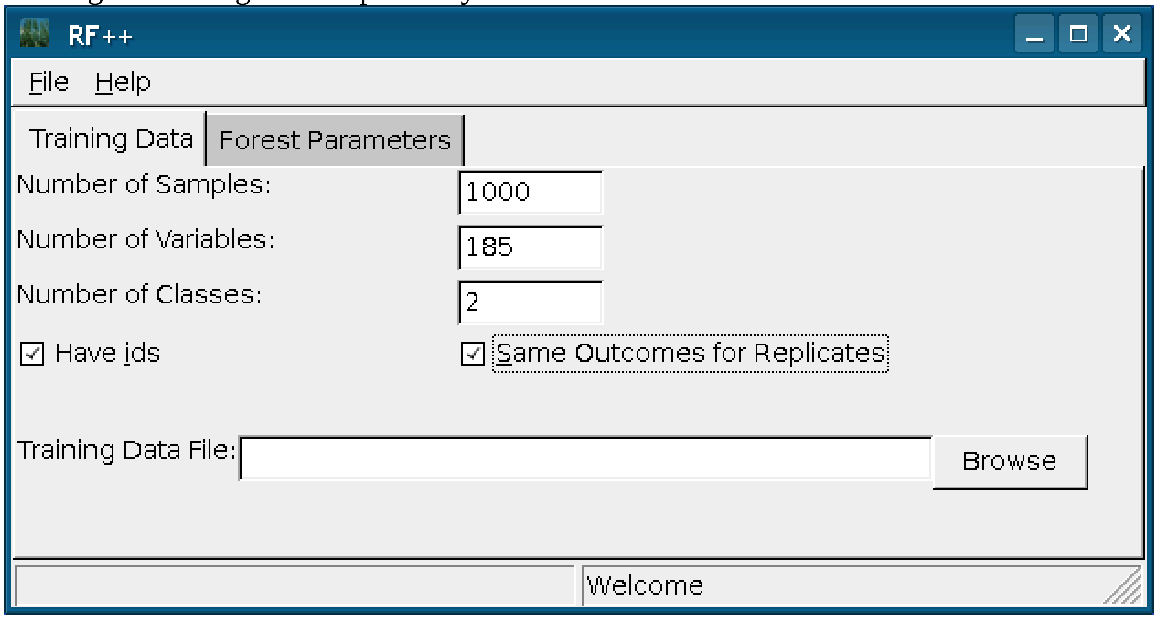

In the ’Training Data’ tab, information describing the dataset must be entered: number of samples in the file, number of variables and number of classes.

Samples in the training data file must be organized in rows (1 row per sample). Columns must be arranged as such: an ID is required in the in the first column if the data is clustered, this ID is optional for non-clustered data. Next, ’Number of Variables’ values for each variable. The last column must be the outcome (classification) column. Note, if IDs are present, RF++ will do subject-level bootstrapping based on the values in the ID columns, where all samples with matching IDs belong to the same subject.

If the data are not clustered then subject-level bootstrapping consists of subject clusters of size one. Thus, in this case subject-level bootstrapping is equivalent to sample-level bootstrapping.

- The 'Number of Variables' field is the number of variables in each sample and must not include the IDs and outcomes columns.
- Outcomes (classifications) are integer values in the range [1,…, number of classes].
- IDs are integers and are used primarily to identify clusters within the data. (Note, no double or character values are allowed).

If the data does not comply with the standard, RF++ will output an error message in the status bar located at the bottom of the GUI window.

Forest Parameters

To generate a forest specify forest parameters by clicking on the 'Forest Parameters' tab.

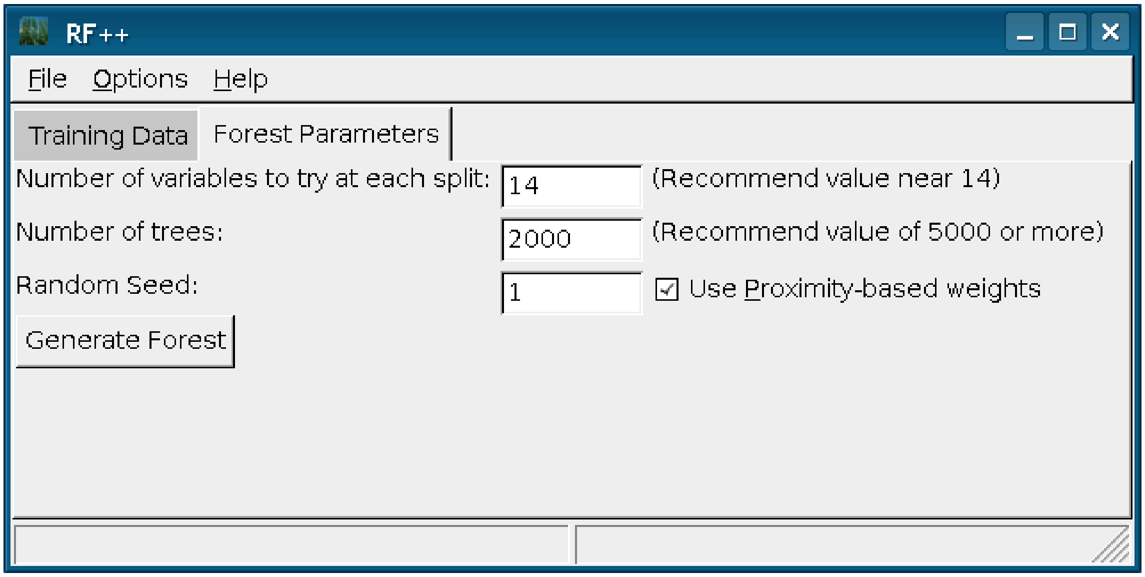

The 1st parameter 'Number of variables to try at each split' will be automatically filled in with the square root of the number of variables entered in the 'Training Data' tab. This default value prevents overfitting the forest to the training data. The user can experiment with different values, but should not increase this number too much or overfitting may occur.

The next parameter is 'Number of trees' to grow. For best results, it is advised to grow between 2,000 and 10,000 trees. Smaller values can be used for quick experimentation with RF++, but larger numbers of trees should be used for effective analysis.

The 'Random Seed' parameter is used to seed the pseudo-random number generator. This value is useful when reproducing the results of prior experiments.

Proximity-based weights can be used for cluster-correlated data with the same replicate outcomes within a subject. This can be done by selecting the 'Proximity-based weights' checkbox.

When all fields have been filled, the forest can be grown by clicking the 'Generate Forest' button.

The progress report, including any error messages, will be displayed in the status bar at the bottom of the RF++ window frame. A 'Error reading in training samples' message may appear if the data parameters are improperly specified. This usually indicates a mismatch of the specified numbers of variables and/or samples with the corresponding values read from the training file. The progress report will change from 'Growing Forest…' to 'Calculating Statistics' and finally to 'Done'.

Training Results

After a forest is built, the Out-of-Bag (OOB) statistics are computed and 3 new tabs appear: 'Training Error', 'Variable Importance' and 'Testing/Predicting Data' tabs.
The 'Training Error' tab displays OOB sample-level and subject-level (when applicable) error rate(s) and confusion matrices.

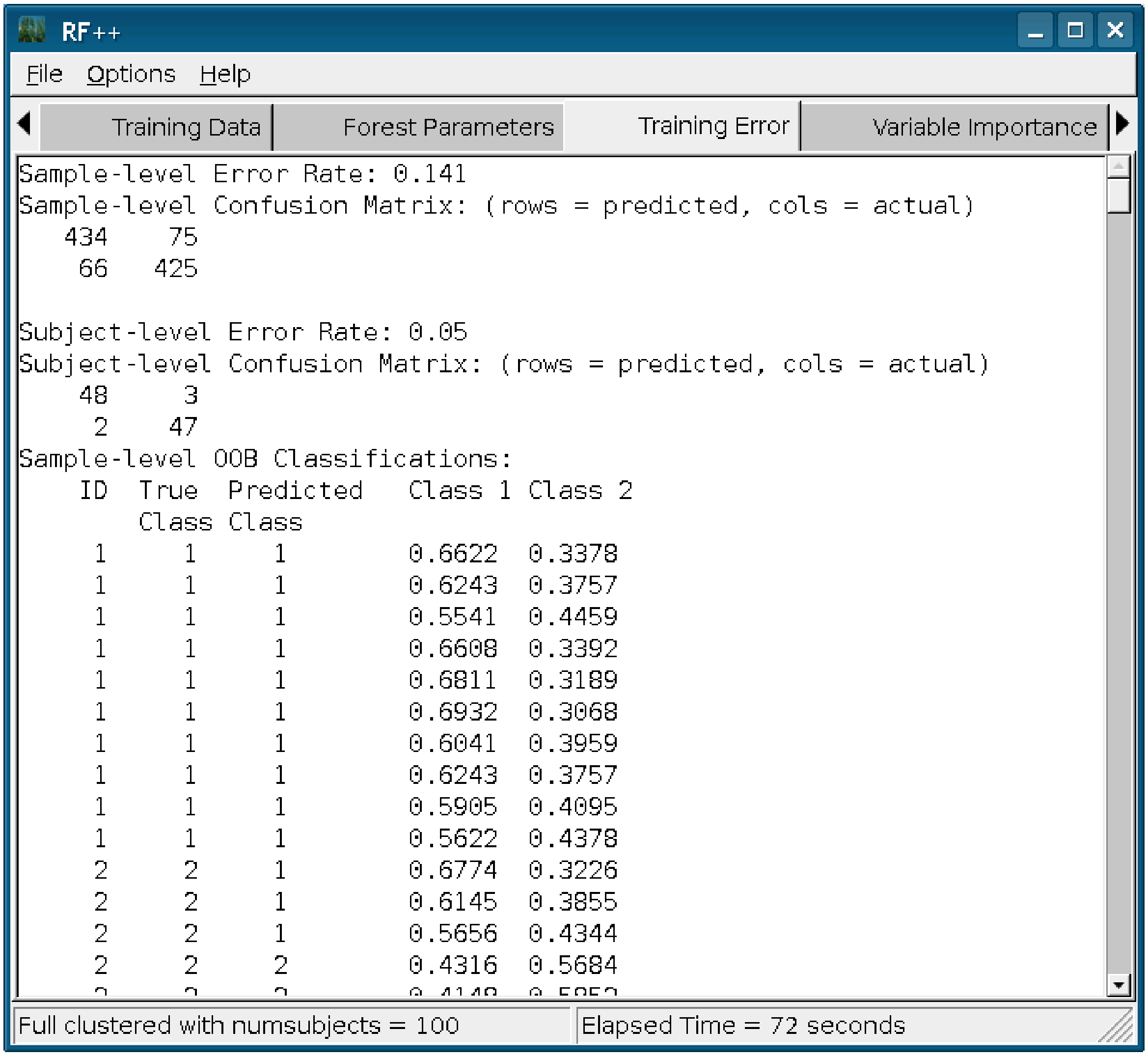

The 'Variable Importance' tab displays variable importance scores for 2 variable importance measures side-by-side. Variables in each column are sorted in decreasing order of importance.

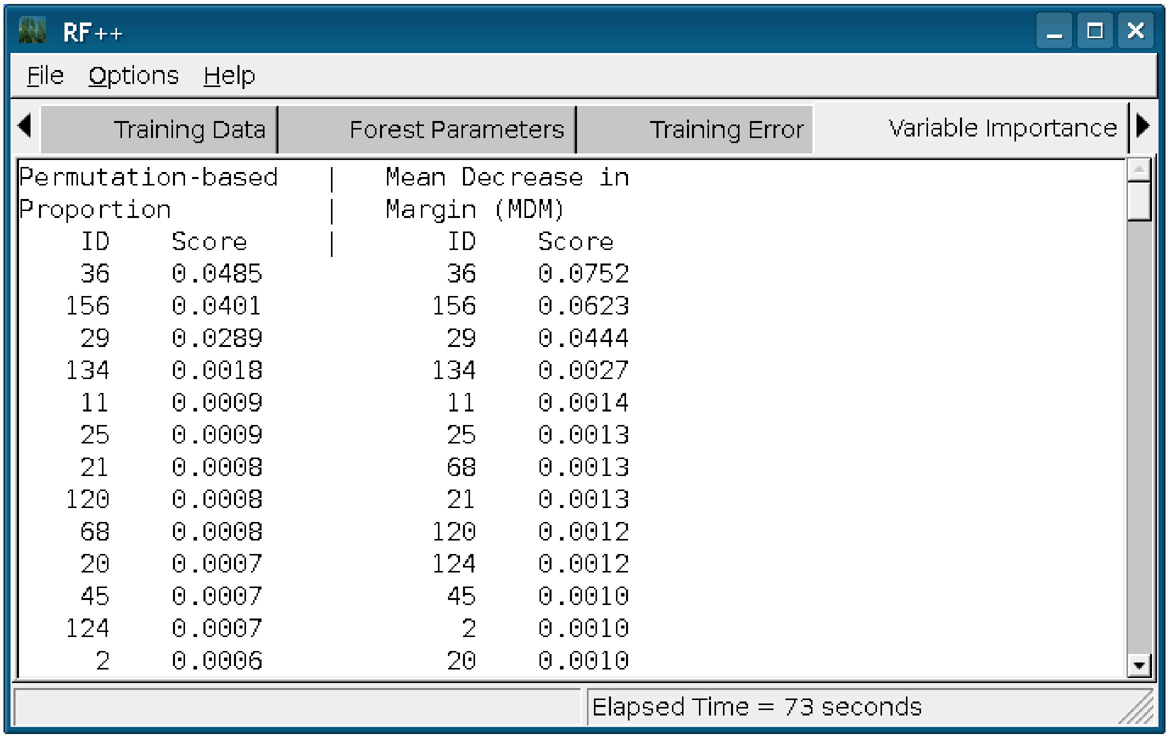

Testing a Forest and Making Predictions

The 'Training/Prediction Data' tab is used to test the performance of the trained forest or to make predictions for unknown cases. This tab is similar to the 'Training Data' tab, but the 'Number of Variables' and 'Number of Classes' fields are automatically filled in form the training dataset and are unchangeable (*greyed out*). The user needs to provide the number of samples in the testing/prediction data file and check the 'Have outcomes' checkbox if the column of outcomes is present in the file. This column should be present only in the testing file (not in the file where prediction of the unknown cases are to be made). Note that, this column is never included in the count of variables.

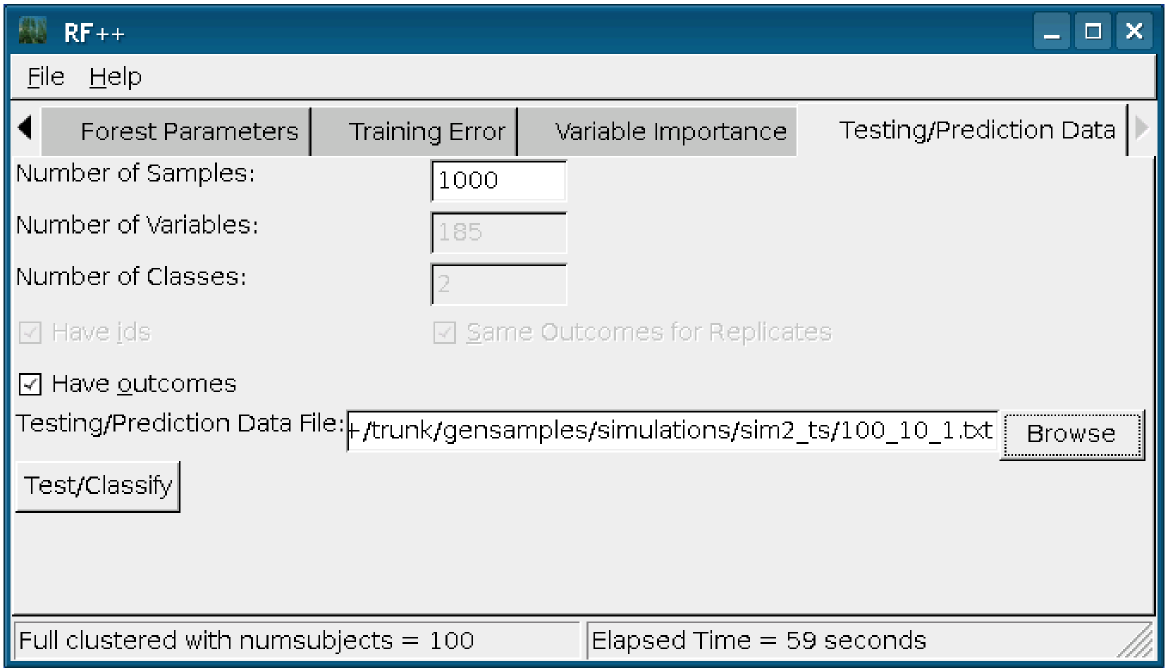

Classifications are displayed in the ‘Testing/Prediction Classifications’ tab. When testing, sample classifications are displayed first, then sample error rate and the sample confusion matrix. If subject-level classification is appropriate, i.e. the data is cluster-correlated and outcomes for each subject replicates belong to the same class, the subject-level classifications, then the subject error rate and subject confusion matrix are displayed.

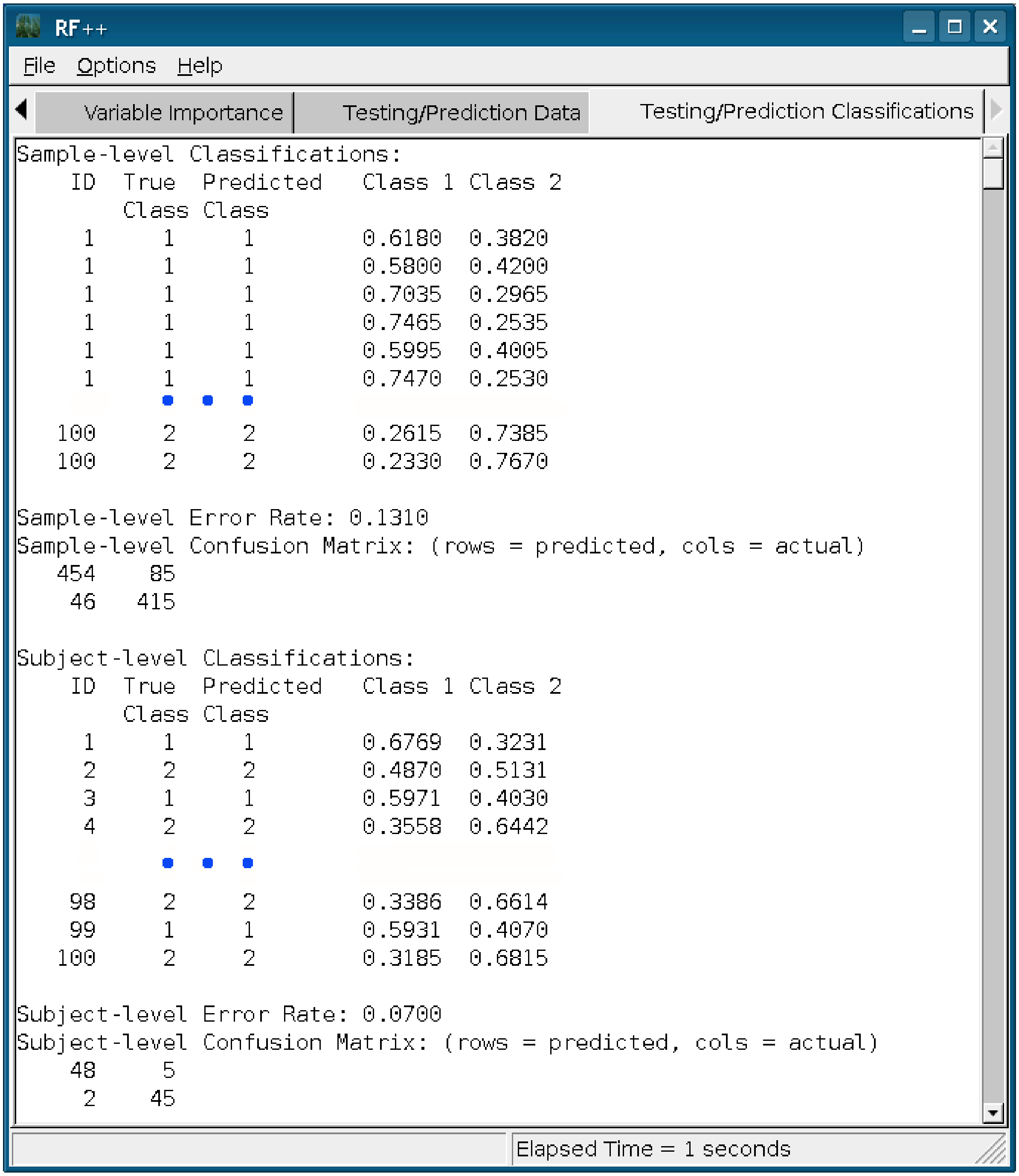

The following columns are displayed: sample/subject IDs, true class, predicted class, followed by the proportions of votes for each of the classes.

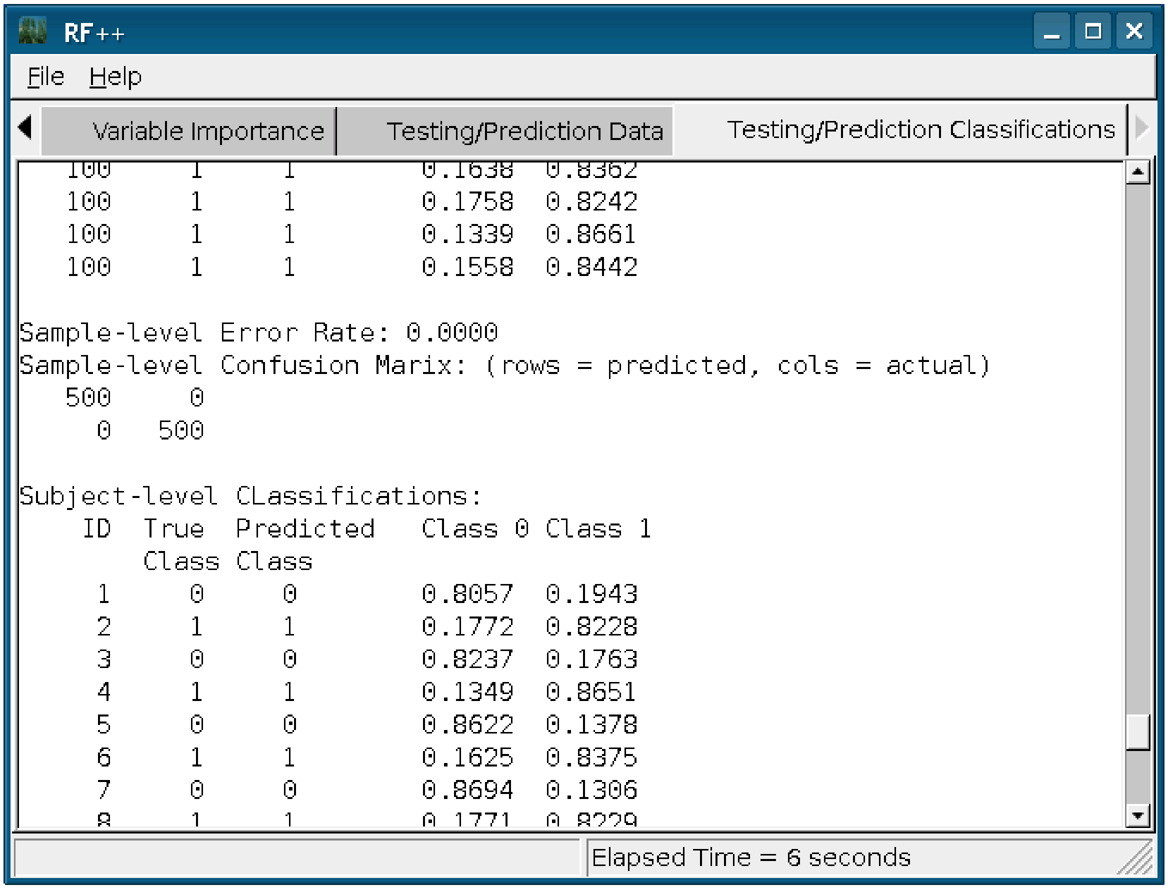

When making predictions, a true outcome column should not be present. Error rate(s) and confusion matrices will not be computed.

Saving forest, variable importance scores and classifications

To save a forest, select on of the following tabs: 'Training Data', 'Forest Parameters', or 'Training Error', then select the 'Save' menu item from the 'File' pull down menu. Forests are saved with the ".rff' file extension. Training error and confusion matrices are also saved in this file.

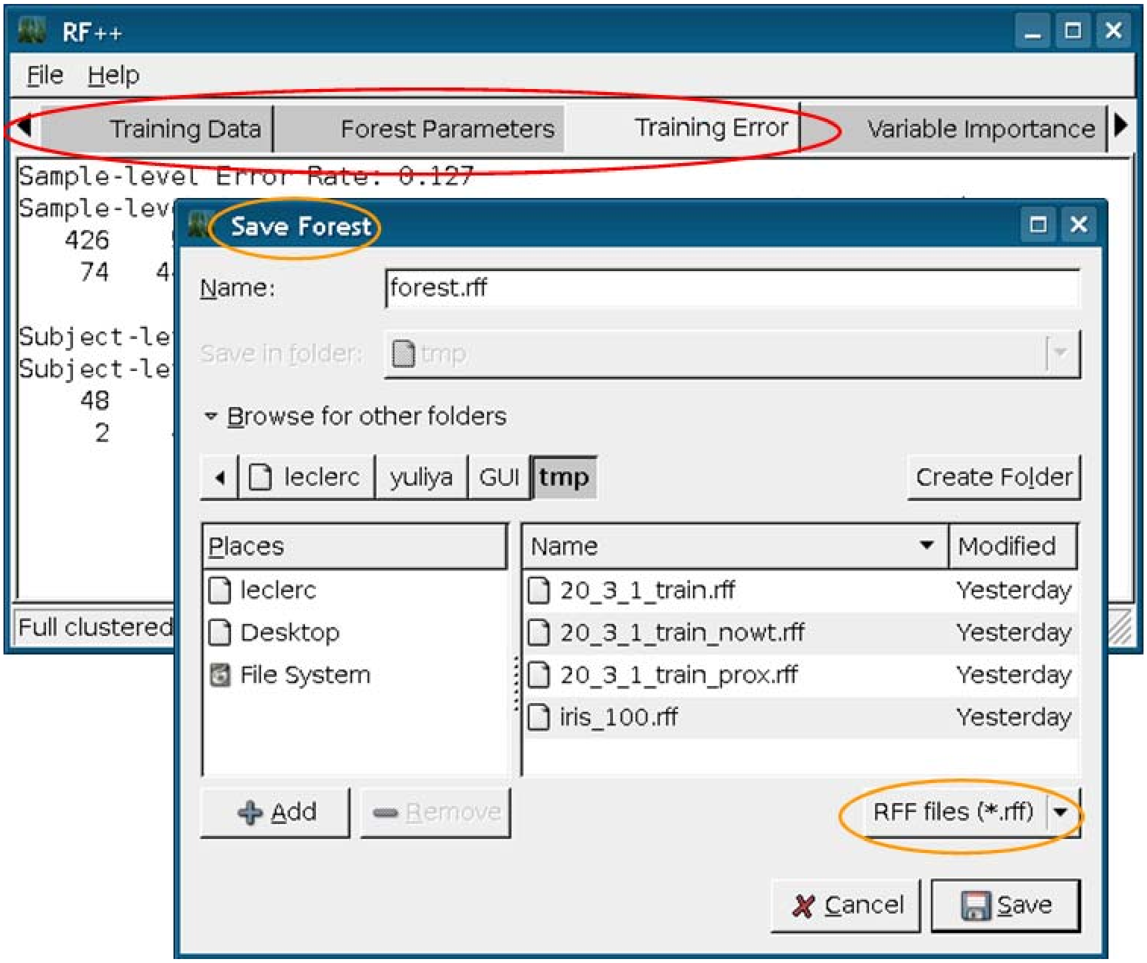

Variable Importance measures are saved by first clicking on the 'Variable Importance' tab and then selecting the 'Save' menu item from the 'File' pull down menu. This file is identical to the text displayed to the user in RF++ window.

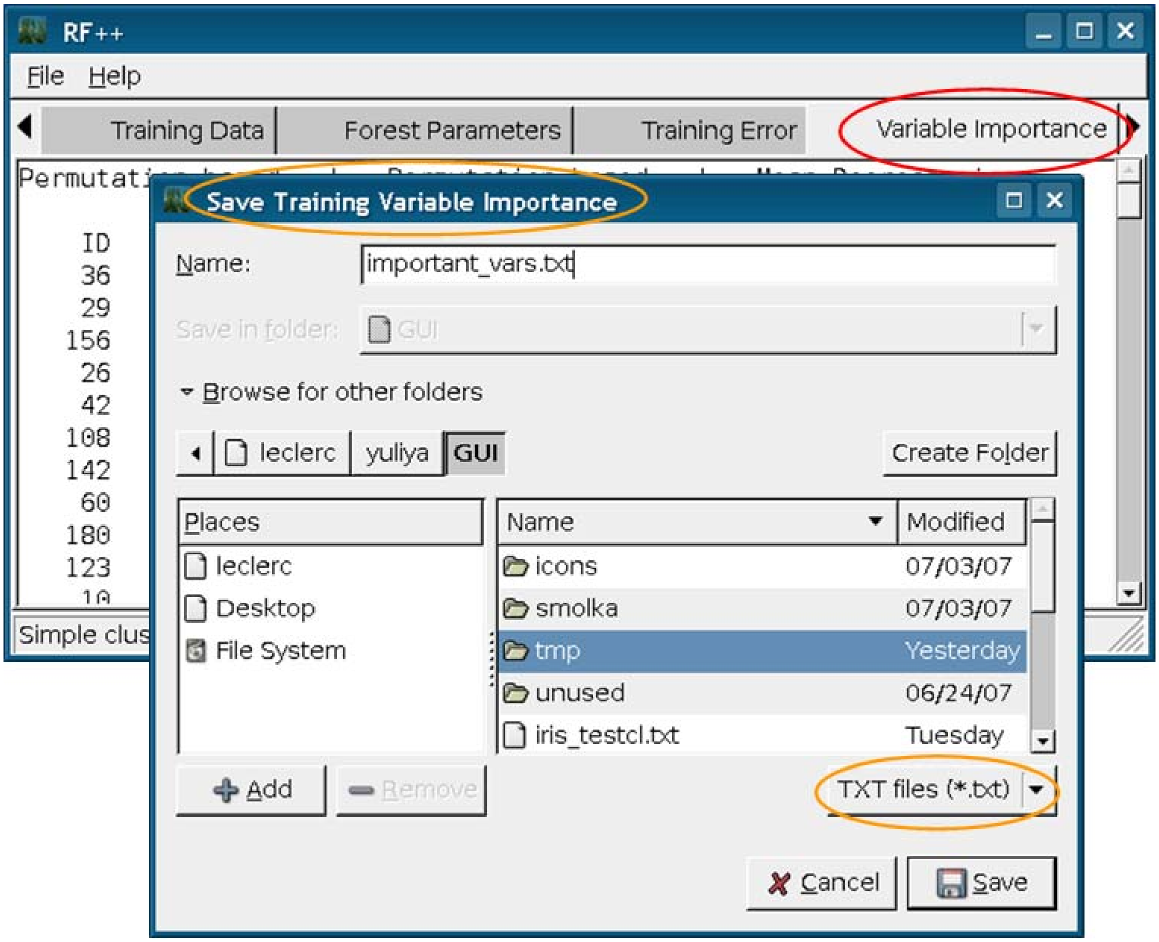

Classifications/predictions can be saved by first clicking on the 'Testing/Prediction Classifications' tab and then selecting the 'Save' menu item from the 'File' pull down menu from the.

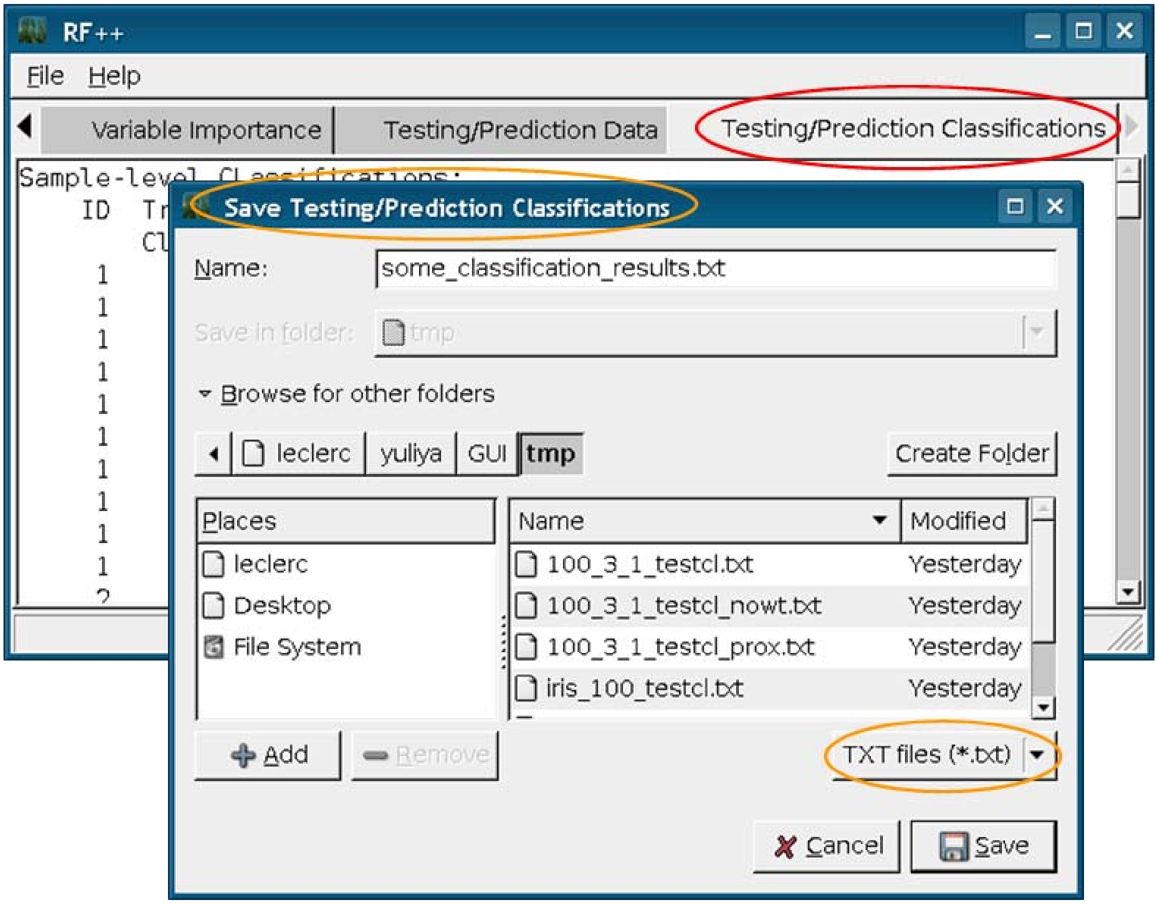

Loading an Existing Forest

A previously forest can be loaded by selecting the 'Open' menu item from the 'File' pull down menu. After a forest is loaded, 4 tabs that describe the forest are displayed. The first 3 tabs describe the forest: 'Training Data', 'Forest Parameters', and 'Training Error'. The 'Testing/Prediction Data' tab is opened for the user to specify testing or prediction dataset parameters in order to test performance of the forest or to make predictions for unknown cases.

Forest File Description

XML File

The XML schema for a forest is defined in the file 'forest.xsd'. This schema defines a forest consisting of forest attributes such as number of samples used to grow a forest, number of trees, etc. followed by a sequence of trees. Each tree is a sequence of nodes. Nodes are listed in level-order, though knowledge of this ordering is unnecessary for parsing.

The correct linkage structure of a tree can be determined by using the node attribute 'id' which uniquely identifies a node. Each non-terminal node also contains ids of the left and right children nodes. An example of a forest in XML format is listed in the supplementary file 'forest.xml'.

Text File

The forest is saved as a text file with the '.rff' extension. It is best to read the descriptions provided for the XML file syntax, files 'forest.xsd' and 'forest.xml'. The '.rff' file contains more information then the XML file, such as the confusion matrices that is useful when a trained forest is loaded into RF++ GUI. An example is available in the supplementary file 'forest.rff'.

Example:

```
150 4 0 0 3
2
1234567
100
50 3 1.6 150 -1
43.7579 2 1.9 95 -1
55 -1 0 55 2
45 -1 0 45 0
42.64 2 4.9 50 -1
46 -1 0 46 1
4 -1 0 4 2

50 2 4.8 150 -1
47.3958 3 0.6 96 -1
50.1481 3 1.7 54 -1
39 -1 0 39 0
53.1404 0 4.9 57 -1
5 2 5 8 -1
46 -1 0 46 2
1 -1 0 1 2
54.0357 3 1.6 56 -1
1.66667 1 2.2 3 -1
5 -1 0 5 2
55 -1 0 55 1
1 -1 0 1 2
1 -1 0 1 2
2 -1 0 2 1
```

150 4 0 0 3
2
4
50 3 0.6 150 -1
46 -1 0 46 0
53.2308 3 1.7 104 -1
52.5077 2 4.9 65 -1
35.2051 0 5.9 39 -1

… (the rest of the trees)

The numbers in the file are as follows:
150 4 0 0 3
number_of_samples number_of_variables have_ids clustered_outcomes number_of_classes
have_ids – 0/1 - 0 if no IDs were present in the training data, 1 if IDs were present.
clustered_outcomes – 0/1 – 0 if training data is not clustered and/or outcomes were not the same for all replicates within a subject. 1 if training data was clustered with outcomes for all replicates within a subject belonging to the same class. If this is 1, then subject-level classifications and error rates are produced in addition to the sample-level classification and error rate.

2
number_of_variables_to_split_at_each_node

1234567
random seed

100
number_of_trees

50 3 1.6 150 -1
Gini_score split_variable split_variable_value sample_size_reached_this_node class
Gini score is used to decide which variable and its value will produce the best separation of training data into distinct classes. Sample size that reached a particular node is printed for purposes of seeing how the splits are made and how many samples a split separates from the rest of the samples in a particular node. The 1st node is the root node that has all the samples, so we see the total number of the samples – 150, which is the same as in the 1st line of the file.
Variables are numbered 1,2,3,… Class (last value in the line) is in the range 1,2,… for terminal nodes (nodes that produce classifications) and -1 for nodes inside the tree that do not produce the classification and are split further.
Trees are separated by a new line. After all the trees are written out more forest information is added:

…
0 4
1 1 1 1
50 7 4
0 41 5
0 2 41

0
<End of file>
----

0 100
0 - which_tree_weight weight_vector_size
which_tree_weight – 0/1, 0 if no tree weights were used, 1 if proximity-based weights used
100 - weight_vector_size – number equal to the number of trees in the forest as each tree has a weight

1 1 1 1 … 111
Tree weights, as one long line with number of trees values in it. Here the weights are set to 1 and thus do not affect the voting.

50 0 4
0 46 2
0 4 48
Sample-level confusion matrix. Matrix shows the numbers of the Out-of-bag (OOB) samples classified into each of the classes. Rows are the predicted outcome, columns are true outcome. Values on the diagonal are the correct classifications, all values off diagonal were classified incorrectly as the class in different row. Class labels are not printed here, classes are ordered as class1,2,… in rows and columns.

0
subject-level_confusion_matrix identifier (flag) – 0/1, 0 if no subject-level confusion matrix follows, 1 if subject-level confusion matrix printed.

Forest file output with the subject level confusion matrix

More information is stored in the forest file if subject-level classification is done. In the following example 2 classes were available.

. . .
2 100
0.516667 0.433333 0.433333 0.4 0.513333 0.39 0.39 0.39 0.546667 0.433333   . . .   0.576667
122   36
28   114

1
43 11
7   39
<End of file>
----
2 100
2 – proximity based weights are used
100 – number of trees in the forest (small for the purpose of the example)

0.516667 0.433333 0.433333 0.4 0.513333 0.39 0.39 0.39 0.546667 0.433333 . . . 0.576667
tree weights, 100 weights

122 36
28 114
sample-level confusion matrix

1
flag (indicator) if subject-level confusion matrix follows, 1 means subject level matrix is present
43 11
7   39
subject-level confusion matrix

Generally, the users do not need to be familiar with the syntax of these files. RF++ will save and load these files automatically. If someone wants to further investigate the rules used to grow trees and consequently a forest the ".rff" and XML files will provide valuable insight into the rules.
